# Detection of aryl hydrocarbon receptor agonists in human samples

**DOI:** 10.1038/s41598-018-23323-4

**Published:** 2018-03-21

**Authors:** Veit Rothhammer, Davis M. Borucki, Jessica E. Kenison, Patrick Hewson, Zhongyan Wang, Rohit Bakshi, David H. Sherr, Francisco J. Quintana

**Affiliations:** 1 Ann Romney Center for Neurologic Diseases, Brigham and Women’s Hospital, Harvard Medical School, Boston, MA USA; 2 Dept. of Environmental Health, Boston University School of Public Health, Boston, MA USA; 3 Broad Institute of MIT and Harvard, Cambridge, MA USA

## Abstract

The aryl hydrocarbon receptor (AHR) is a ligand-activated transcription factor with important functions in the immune response and cancer. AHR agonists are provided by the environment, the commensal flora and the metabolism. Considering AHR physiological functions, AHR agonists may have important effects on health and disease. Thus, the quantification of AHR agonists in biological samples is of scientific and clinical relevance. We compared different reporter systems for the detection of AHR agonists in serum samples of Multiple Sclerosis (MS) patients, and assessed the influence of transfection methods and cell lines in a reporter-based *in vitro* assay. While the use of stable or transient reporters did not influence the measurement of AHR agonistic activity, the species of the cell lines used in these reporter assays had important effects on the reporter readings. These observations suggest that cell-specific factors influence AHR activation and signaling. Thus, based on the reported species selectivity of AHR ligands and the cell species-of-origin effects that we describe in this manuscript, the use of human cell lines is encouraged for the analysis of AHR agonistic activity in human samples. These findings may be relevant for the analysis of AHR agonists in human samples in the context of inflammatory and neoplastic disorders.

## Introduction

The aryl hydrocarbon receptor (AHR) is a ligand-activated transcription factor which was initially described as a molecular sensor for environmental toxins^1^. However, it is now clear that AHR is activated not only by environmental toxins, but also by commensal and endogenous agonists which can initiate ligand-, cell- and tissue-specific biologic responses. Indeed, the role of AHR in inflammatory asnd neoplastic diseases has been studied extensively^1–9^. AHR plays an important role in the control of T cells, dendritic cells, gut intraepithelial lymphocytes, tumor cells and astrocytes^1–9^. For example, we recently reported that AHR activation in astrocytes limits inflammation in the central nervous system (CNS) during experimental autoimmune encephalomyelitis (EAE), an animal model of multiple sclerosis (MS)^8^. Tryptophan (Trp) metabolites produced by commensal bacteria in cooperation with host enzymes activate AHR in astrocytes to limit CNS inflammation^8^. Interestingly, we detected decreased AHR activation in MS brain lesions as indicated by the expression of the AHR target gene *CYP1B1*, concomitant with decreased levels of AHR agonists in MS sera detected using a cell-based reporter assay and mass spectrometry^8^. Moreover, AHR agonistic activity in sera from MS patients was correlated both with disease activity and more severe stages of MS^7^. Collectively, these data identify AHR in astrocytes as a negative regulator of CNS inflammation. Interestingly, decreased AHR agonists were also reported in inflammatory bowel disease (IBD) patients^4^, suggesting that deficits in AHR-driven immune regulation may contribute to multiple human disorders.

AHR agonistic activity is detected in clinical and commercial human samples^10–18^. Considering the multiple biological roles of AHR, the quantification of agonists in clinical samples may be of interest to identify potential associations of this pathway with human disorders. However, the total AHR agonistic activity as determined with reporter assays reflects the net activity, which integrates multiple variables such as the levels of AHR agonists of diverse origins (environmental, commensal, dietary and metabolic), their uptake, activation and degradation, as well as the balance between AHR agonists, yet to be characterized physiologic AHR antagonists and other molecules that modulate AHR activation indirectly through their effects on other signaling pathways. Here, we compared reported assays available for the detection of AHR agonists with the goal of guiding future studies of AHR agonistic activity in biological samples.

## Results

### The species of the cell lines used influence the detection of AHR agonists in reporter assays

**Figure 1:**
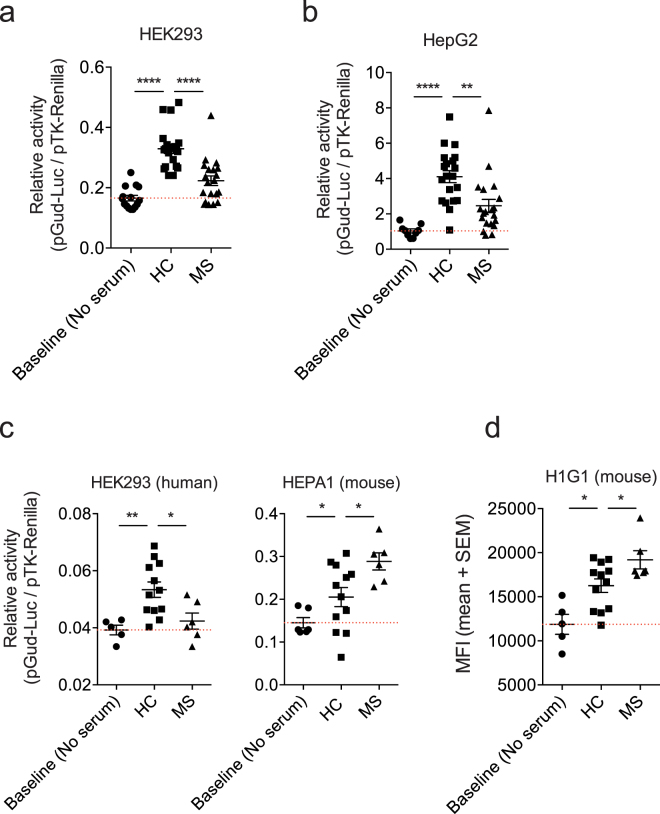
AHR agonistic activity in human sera is influenced by the species of the cell line used in the reporter assay. (**a**) Human embryonic kidney cells (HEK293) were transfected with pGud-Luc1.1 and pTK-Renilla. One day after transfection, cells were incubated with human serum from healthy controls (HC) or MS patients (MS) and luciferase activity was assessed after 24 hours. Control cultures to which no serum was added were used to determine baseline activity. **(b**,**c)** Luciferase assay performed as in (a) using human HepG2 (**b**) or mouse HEPA1 **(c)** cell lines. **(d)** A stable AHR reporter mouse H1G1.1C3-Luc cell line was used to measure AHR ligand activities 24 hours after incubation with HC or MS serum. FACS analysis of mean fluorescence intensity (MFI) for reporter expression. *P < 0.05, **P < 0.01 and ***P < 0.001 by one-way analysis of variance (ANOVA) followed by Tukey’s post-hoc test.

A method for the detection of AHR agonists is the use of cell lines transfected (stably or transiently) with a reporter plasmid containing an AHR responsive promoter element (XRE) which is activated upon ligand-induced AHR recruitment and thereafter drives the expression of reporter molecules such as firefly luciferase or green fluorescent protein (GFP). Thus, as an initial approach to compare different reporter systems we first analyzed the impact of the cell line used in AHR reporter assays. To this end, we transfected human HepG2 hepatoma cells and human HEK293 cells transiently with construct encoding a firefly luciferase under the control of four AHR responsive elements (pGud-Luc1.1)^19^. In both cell lines, we detected decreased AHR agonist activity in MS sera when compared to healthy controls (Fig. 1a,b). Please note that patient treatment status did not affect the AHR agonistic activity detected (Supplemental Fig. 1 and Table 1). Thus, the detection of decreased AHR agonistic activity in MS sera does not depend on the use of a specific human cell line.

**Table 1:** Characteristics of MS patients and controls.

Figure	Cohorts	Females	Age	Disease duration	EDSS	Treatment
1a,b,2b	Controls (20)	14 (70.0%)	27.5 [26.0: 34.8]	none	none	none
	RRMS (20)	16 (80%)	48.2 [40.7: 56.2]	14.8 [12.2: 20.1]	1.5 [1.0: 2.0]	5 (25.0%)
1c,d	Controls (12)	8 (66.7%)	30.5 [28.0: 38.0]	none	none	none
	RRMS (6)	5 (83.3%)	39.1 [36.0: 40.7]	14.7 [8.3: 15.8]	1.5 [0.75: 2.0]	2 (33.3%)
3a,b	Controls (20)	17 (85.0%)	57.0 [45.5: 74.5]	none	none	none
	CIS (14)	9 (60%)	32.5 [27.9: 34.5]	Onset	1.7 [1.4: 2.0]	0 (0%)
	RRMS (40)	26 (65%)	33.0 [27.3: 39.8]	1.1 [0.5: 3.9]	1.8 [1.5: 2.5]	2 (5%)
3c	RRMS (9)	7 (77.8%)	36.0 [29.5: 39.5]	2.3 [0.9: 4.3]	2.0 [1.5: 3.0]	0 (0%)

**Figure 2:**
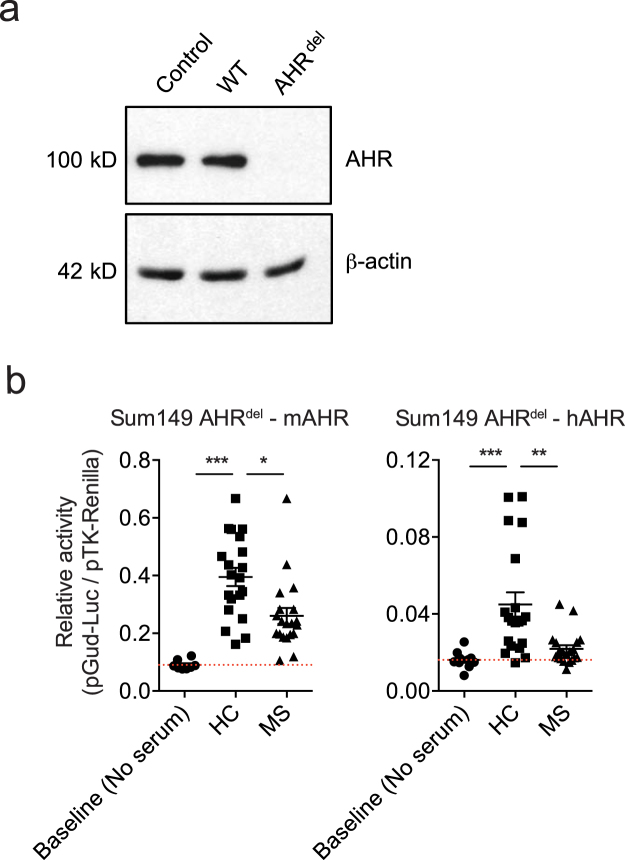
Cell lines species, but not AHR origin, influence AHR agonistic activity. (**a**) Representative immunoblot showing AHR and β-actin protein levels in wildtype (WT) SUM149 cells, CRISPR control cells with Cas9 and CRISPRv2 transduction (Control) and AHR^del^ cells transduced with Cas9 and CRISPRv2-sgRNA containing AHR target sequences (AHR^del^). **(b)** Sum149 AHR^del^ cells deficient for endogenous AHR were transfected with mouse (left) or human (right) AHR expression plasmids and used in the luciferase assays described above. *P < 0.05, **P < 0.01, ***P < 0.001 by one-way analysis of variance (ANOVA) followed by Tukey’s post-hoc test.

**Figure 3:**
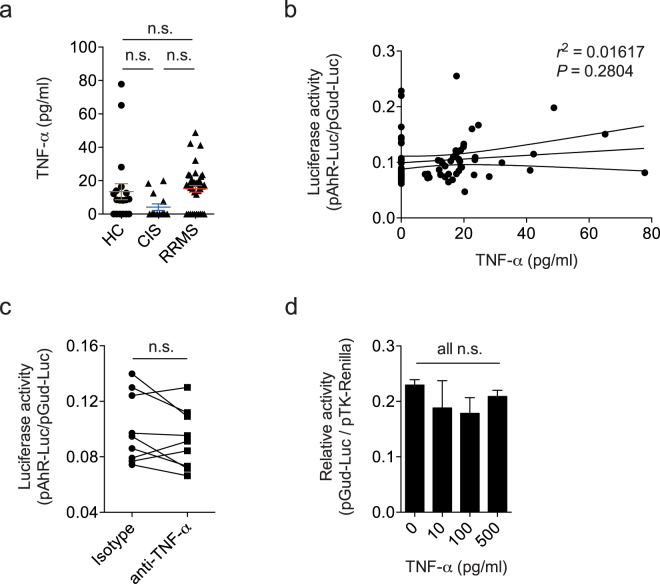
TNF-α does not suppress AHR agonistic activity in MS sera. (**a**) TNF-α was quantified by multiplex ELISA in serum samples from healthy controls (HC), patients with clinically isolated syndrome (CIS), or relapsing/remitting MS (RRMS). **(b**) Linear regression analysis of TNF-α protein levels in human sera and luciferase activity as detected in the transient HEK293 transfection assay system. Dotted lines represent 95% confidence intervals. Numbers indicate R^2^ and *P* value of linear regression analysis. (**c**) MS serum samples were incubated with blocking antibodies to TNF-α or isotype control and added to HEK293 cells transiently transfected with pGud-Luc1.1 and pTK-Renilla. Relative luciferase activity (Firefly luciferase activity/Renilla luciferase activity) was assessed after 24 hours. **(d)** HEK293 cells transiently transfected with pGud-Luc1.1 and pTK-Renilla were incubated with increasing concentrations of TNF-α in the presence of the AHR ligand Kynurenine. In a, c, and d, data are mean ± s.e.m. n.s. not significant as determined by one-way analysis of variance (ANOVA) followed by Tukey’s post-hoc test.

“Females” indicates the absolute number and percentage of females in the group. “Age”, “Disease duration” and “EDSS” are shown as mean, with 25% and 75% percentiles indicated in square brackets. “Treatment” indicates the absolute number and percentage of treated patients. Patients in Figs. 1a,b and 2b were treated with IFN-β (4 patients) or glatiramer acetate (1 patient). Patients in Figs. 1c,d were treated with IFN-β (1 patient) or glatiramer acetate (1 patient). Patients in Figs. 3a,b were treated with IFN-β (2 patients). No additional relevant comorbidities or pharmaceutical treatments were reported in patients or controls. CIS and MS were defined based on established criteria at the time point of diagnosis.

Next, we analyzed the effect of the species of the cell line used in the assay. Again, we detected decreased AHR agonistic activity in MS sera when we used human HEK293 cells transiently transfected with the pGud-Luc1.1 AHR reporter (Fig. 1c, left). However, we measured increased AHR agonistic activity in MS sera when we used transiently transfected mouse liver hepatoma HEPA1 cells (Fig. 1c, right). Similarly, mouse H1G1.1C3 cells stably transfected with an AHR reporter^20^ detected increased AHR agonistic activity in the same set of MS sera (Fig. 1d). Taken together, these data suggest that the species of the cell line used in the reporter assay influences the detection of AHR agonistic activity, and not whether the cells were stably or transiently transfected.

### Cell line species, but not AHR species, affects the quantification of AHR agonistic activity

Structural differences between mouse and human AHR led to the identification of physiological species-specific agonists that activate human but not murine AHR^19,21–24^. Since the transient and stable reporter assays used in these studies are based on the activation of the AHR expressed by the transfected cell, we hypothesized that differences in the sensitivity of mouse and human AHR to agonists in human sera may reflect the activation of human or murine AHR in the cells used in the reporter assay. To address this point, we used human Sum149 breast cancer cells (Sum149 AHR^del^ cells), in which endogenous AHR expression was deleted using CRISPR/Cas9 technology (Fig. 2a). Human Sum149 AHR^del^ cells were co-transfected with the pGud-Luc1.1 AHR reporter plasmid and a construct coding for either mouse or human AHR, and used to analyze AHR agonistic activity. We detected decreased AHR agonistic activity in MS sera analyzed using human Sum149 AHR^del^ cells expressing either murine or human AHR (Fig. 2b). Collectively, these findings suggest that the cell line species, but not the species of AHR itself, affects the quantification of AHR agonist activity in serum.

### TNF-α in MS sera does not interfere with the detection of AHR agonistic activity

TNF-α is reported to inhibit the activation induced by TCDD of an AHR reporter transiently transfected with Lipofectamine through a mechanism mediated by NF-κB^25^. Thus, it is conceivable that TNF-α in clinical samples interferes with the detection of AHR agonist activity. Indeed, TNF-α is reported to be increased in MS sera by some studies^26,27^, but not consistently^28,29^. To determine whether TNF-α suppresses AHR agonistic activity in MS sera, we first quantified TNF-α in healthy control, definite MS and clinically isolated syndrome (CIS) samples using an ELISA system with a lower limit of detection of 3.2 pg/ml. We did not detect a significant increase of TNF-α in the CIS or MS samples we analyzed (Fig. 3a). TNF-α serum levels were also not correlated with AHR agonistic activity detected with cells transiently transfected (Fig. 3b). Furthermore, TNF-α blockade with specific antibodies did not increase the AHR agonistic activity detected in MS sera using HEK293 cells transiently transfected with the AHR responsive reporter construct (Fig. 3c). Conversely, exogenously added TNF-α did not suppress AHR activation when tested on HEK293 cells transiently transfected (Fig. 3d). These findings suggest that the suppression of AHR activation by TNF-α suggested in previous studies does not affect the detection of AHR agonist activity in sera using human HEK293 cells transfected transiently with the AHR-Luciferase construct.

## Discussion

In this study, we analyzed cell-line based reporter assays for the detection of AHR ligands in human serum. Specifically, we analyzed the effect of the transfection method, the cell line species and TNF-α interference in these reporter systems. While the use of stable versus transient transfection did not affect the quantification of AHR agonistic activity in MS sera, the species of the cell line used had significant effects on the determination of AHR agonistic activity. This result was independent of the species of AHR itself. Finally, TNF-α did not impair the measurement of AHR ligands in transiently transfected human HEK293 cells.

It has recently been reported that AHR agonistic activity varies during the course of inflammatory diseases such as MS and inflammatory bowel disease (IBD), suggesting that changes in circulating AHR agonists reflect alterations in the commensal flora, uptake, degradation or metabolism of physiological AHR agonists^4,7,8^. Thus, the quantification of AHR agonists is of clinical interest. Multiple *in vivo* and *in vitro* bioassays exist to quantify AHR agonistic activity in biological samples^30^. Denison and colleagues developed reporter systems based on constructs in which the expression of the firefly luciferase gene is driven by XREs present in the promoter of *Cyp1a1*, a metabolic gene under control of AHR^31^. Upon binding of ligands to AHR, the AHR-ligand complex translocates to the nucleus and binds to the XRE elements, inducing firefly luciferase expression, the levels of which correlate with the net AHR agonistic activity in the sample. Our findings suggest that the species of the cell lines used for these reporter assays may affect the AHR agonistic activity detected in clinical samples. Moreover, these observations suggest that cell-specific factors influence AHR activation and signaling. This is important to consider when using reporter assays to quantify net AHR agonistic activity, which is influenced not only by the production, uptake and metabolism of AHR agonists, but also by AHR antagonists and other modulators of AHR signaling.

Several factors may contribute to the effects of the species of the cell lines used in reporter assays. First, it is theoretically possible that yet uncharacterized molecules in MS sera activate AHR inhibitory pathways in human but not in murine cells. If present, those inhibitors will decrease net AHR agonistic activity in MS sera, limiting AHR-dependent anti-inflammatory mechanisms. Metabolomic approaches^8^ could determine the contribution of specific agonists to the reduced AHR agonistic activity in MS sera. Second, our findings using a human cell line deficient in endogenous AHR reconstituted with mouse or human AHR suggest that species specific mechanisms affect the sensing of AHR agonists irrespective of the origin of AHR itself. These mechanisms may involve factors controlling AHR interactions in the cytoplasm with the 90-kDa heat shock protein (HSP90), the AHR-interacting protein and the cochaperone p23, which have to release AHR upon ligand binding to allow its translocation to the nucleus^1^. Third, interactions of AHR with additional transcription factors such as nuclear factor kappa B (NF-κB) could be cell-line and species specific, influencing AHR binding to XRE elements^32^. Indeed, we evaluated the potential interference of TNF-α in the reporter assay because TNF-α is a known NF-κB activator, and NF-κB and AHR signaling cross-talk at multiple levels^33–35^. TNF- α did not interfere with the detection of AHR ligands in our system, but additional yet unknown serum factors may influence AHR signaling in a species and cell-line specific manner. Taken together, these studies highlight the complexity of measuring AHR agonistic activity in clinical samples. Based on the reported species selectivity of AHR ligands and the cell species effects that we report in this manuscript, the use of human cell lines is encouraged for the analysis of AHR agonistic activity in human samples. Moreover, collectively, these findings further support the use of targeted methods to quantify specific AHR agonists and/or antagonists to study their role in human disease.

## Methods

### Cell lines

HEK293 cells were purchased from ATCC. HepG2 human hepatoma cells and HEPA1 mouse hepatoma cells were obtained from the ATCC. H1G1.1C3-GFP cells, the generous gift of Dr. M. Denison, have been described previously^20^. Sum149 AHR^del^ cells were generated as follows: the CRISPR vector lentiCRISPR v2 (Addgene no. 52961, Cambridge, MA) contains Cas9 and a guide RNA cloning site (*BsmBI*). The two target sequences (5′-CCTACGCCAGTCGCAAGCGG-3′ and 5′-CCGAGCGCGTCCTCATCGCG-3′, NM_001621), selected by CRISPR designer (http://crispr.mit.edu), are located in the first exon of the *AHR*. The construct was confirmed by DNA sequencing. Sum149 cells were infected with AHR lentiCRISPRv2 lentivirus according to the standard protocol^36^. Cells were selected for 10 days with 2.0 μg/ml puromycin. AHR^del^ was confirmed by Western blotting. For western blotting to confirm AHR deletion, AHR antibody was purchased from Cell Signaling Technologies, Danvers, MA (13790#,1:1000 dilution) and β-actin antibody was from Sigma-Aldrich, (A5441,1:2000).

### Transient transfection assay

HEK293, HepG2, and HEPA1 cells were used in the transient transfection system, as previously described^7,8^. In brief, 15,000 cells per well were plated in 96 well flat bottom plates. 24 h after plating, cells were transfected with equal amounts of pGud-Luc1.1^19^, generously provided by Dr. M. Denison, and pTK-Renilla (Renilla luciferase under control of constitutively active Thymidine kinase promoter, Promega) using Fugene-HD Transfection Reagent (Promega) as suggested by the manufacturer. In some experiments Sum149 AHR^del^ cells were plated into 96 well flat bottom plates and transfected with equimolar amounts of pGud-Luc1.1, pTK-Renilla, and mouse or human AHR expression plasmids. After 24 h, transfected cells were incubated with DMEM supplemented with 10% of patient serum in duplicates. Luciferase activity was analyzed 24 h later using the Dual Luciferase Reporter System (Promega). Firefly luciferase activity was normalized to Luciferase activity to determine relative AHR agonistic activity. The study was approved by the Institutional Review Board of Brigham and Women’s Hospital and all participants provided written informed consent. All methods were performed in accordance with the relevant guidelines and regulations. In some experiments, MS sera were incubated with a blocking antibody to TNF-α (2 μg/ml, R&D Systems, AF-210) or isotype control (2 μg/ml, R&D Systems, AB-108) for 30 mins and used in luciferase assay as described above. For TNF-α titration experiments, the AHR ligand Kynurenine (2 µM) was mixed with increasing concentrations of human TNF-α (R&D Systems, 210 TA/CF).

### Stable transfection assay

H1G1.1C3-GFP cells were plated into 96 Well flat bottom plates. After 24 hours, cells were incubated with patient or control serum in duplicates as described for the transient transfection system. 24 hours later, medium was removed, cells washed with PBS, trypsinized and transferred to 96 Well round bottom plates. Mean fluorescence intensities of GFP^+^ cells were assessed by flow cytometry using a MACSQuant flow cytometer (Miltenyi Biotech) and analyzed in FlowJo.

### Detection of TNF-α

TNF-α in human sera was quantified using the Human 11-Plex Cytokine Kit as recommended by the manufacturer (Cat. No. LHCY-20110S, AYOXXA, Boston, MA).

### Samples

All serum samples were collected and stored at −80**°**C using a standardized protocol as described^37–40^. Clinical characteristics of the cohorts are shown in Table 1.

### Statistical analysis

Statistical analyses were performed with Prism software (GraphPad), using the statistical tests indicated in the individual figure legends. No samples were excluded. The investigators were blinded as to sample cohorts when performing AHR ligand level measurement and samples were run in duplicates. *P* values of <0.05 were considered significant. All error bars represent s.e.m.

## Electronic supplementary material

Supplementary Information
