# Supplementary material for: Detection of aryl hydrocarbon receptor agonists in human samples
Source: Sci Rep. 2018 Mar 21;8:4970. doi: 10.1038/s41598-018-23323-4 (PMC5862868; doi:10.1038/s41598-018-23323-4)
Supplement: Supplementary file 1 — Supplementary Information [file 41598_2018_23323_MOESM1_ESM.pdf]

Detection of aryl hydrocarbon receptor agonists in human samples

Veit Rothhammer[1], Davis M. Borucki[1], Jessica E. Kenison[1], Patrick Hewson[1], Zhongyan Wang[2], Rohit Bakshi[1], David H. Sherr[2] and Francisco J. Quintana[1,3]*

[1]Ann Romney Center for Neurologic Diseases, Brigham and Women's Hospital, Harvard Medical School, Boston, MA, USA.

[2]Dept. of Environmental Health, Boston University School of Public Health, MA, USA

[3]Broad Institute of MIT and Harvard, Cambridge, MA, USA.

*Corresponding author:

Ann Romney Center for Neurologic Diseases

Department of Neurology, Brigham and Women's Hospital, Harvard Medical School

60 Fenwood Road, Boston, MA 02115, USA.

Tel.: +1 617 525 5317; Fax: +1 617 525 5305

Email: fquintana@rics.bwh.harvard.edu

Supplementary information

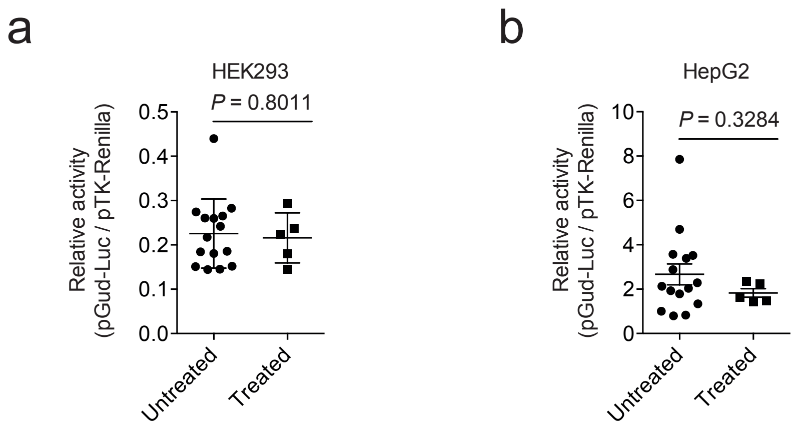
a
HEK293
P = 0.8011
0.5
0.4
0.3
0.2
0.1
0.0
Relative activity
(pGud-Luc / pTK-Renilla)
Untreated
Treated
b
HepG2
P = 0.3284
10
8
6
4
2
0
Relative activity
(pGud-Luc / pTK-Renilla)
Untreated
Treated

Supplementary Figure 1. Stratification of RRMS cohort by treatment. Luciferase activity in patients shown in **Figs. 1a,b** displayed based on the presence or absence of disease modifying therapy. Statistical analysis was performed using Student's *t*-test.
